# Unusual Manifestations of Primary Pancreatic Neoplasia

**DOI:** 10.3390/cancers17193240

**Published:** 2025-10-06

**Authors:** Emilia Włoszek, Kamila Krupa, Marta Fudalej, Hanna Miski, Anna M. Badowska-Kozakiewicz, Andrzej Deptała

**Affiliations:** 1 Students’ Scientific Organization of Cancer Cell Biology, Department of Oncology Propaedeutics, Medical University of Warsaw, 01-445 Warsaw, Poland; emilia.wloszek@student.wum.edu.pl (E.W.); kamila.krupa@student.wum.edu.pl (K.K.); s090695@student.wum.edu.pl (H.M.); 2 Department of Oncology Propaedeutics, Medical University of Warsaw, 01-445 Warsaw, Poland; marta.fudalej@wum.edu.pl (M.F.); anna.badowska-kozakiewicz@wum.edu.pl (A.M.B.-K.); 3 Department of Oncology, National Medical Institute of the Ministry of the Interior and Administration, 02-507 Warsaw, Poland

**Keywords:** new-onset diabetes mellitus, pancreatic ductal adenocarcinoma, depression, pain in cancer, paraneoplastic syndrome, early detection

## Abstract

**Simple Summary:**

Pancreatic ductal adenocarcinoma (PDAC) represents a malignancy characterized by one of the lowest survival rates, and at the time of diagnosis, the majority of tumors are deemed unresectable. Consequently, it is imperative to investigate the early signs and symptoms while conducting screenings for patients at risk of developing PDAC. This approach may provide an opportunity to enhance treatment outcomes. A review of the recent literature focusing on symptoms that arise prior to the diagnosis of PDAC was undertaken, emphasizing the underlying biological mechanisms and potential applications for screening. Furthermore, the role of preexisting pain, depression, diabetes mellitus, and paraneoplastic syndromes in influencing treatment and outcomes was meticulously examined.

**Abstract:**

Pancreatic ductal adenocarcinoma (PDAC) represents a malignancy characterized by one of the lowest survival rates; furthermore, at the time of diagnosis, the majority of tumors are deemed unresectable. Consequently, there exists a pressing need to investigate early signs and symptoms, as well as to implement screening protocols for patients at risk of developing PDAC. By doing so, we may enhance the potential for improved treatment outcomes in light of the typically poor prognosis associated with PDAC. A review of recent literature focused on symptoms that manifest prior to the diagnosis of PDAC has been conducted, emphasizing the underlying biological mechanisms and potential screening applications, alongside data pertaining to the influence of these symptoms on prognosis and treatment. Additionally, the roles of pre-existing pain, depression, diabetes mellitus, and paraneoplastic syndromes in treatment and outcomes were scrutinized to ascertain the feasibility of integrating these factors into clinical practice.

## 1. Introduction

Pancreatic cancer (PC) is classified as a malignant tumor characterized by the lowest survival rate, which is approximately 13%. It ranks as the sixth leading cause of cancer-related mortality globally [1]. The most prevalent histological subtype of this neoplasm is pancreatic ductal adenocarcinoma (PDAC) [2]. Upon the manifestation of symptoms, the lesion is typically deemed unresectable, with the majority of patients presenting with either locally advanced or distant metastatic disease (80–85%) [3]. The early detection of PDAC would significantly enhance overall survival (OS); however, the relatively low prevalence of PDAC renders population-wide screening purposeless [4]. The predominant challenge associated with early detection is identifying individuals at risk within the general population who would derive significant benefits from longitudinal surveillance programs. It is recognized that certain small patient cohorts exhibit an elevated risk for PDAC, particularly individuals possessing inherited genetic mutations or a prior history of pancreatitis. [4]. Another group at risk that we will elaborate upon is elderly patients diagnosed with new-onset diabetes mellitus (NOD) [5]. However, it is necessary to identify additional factors that may contribute to the early diagnosis and treatment of PDAC, including initial symptoms, biomarkers, and other relevant elements.

This review examines the potential initial symptoms of PDAC, specifically diabetes and depression. Furthermore, it explores the role of pain and its perception during the early stages of the disease, as well as its impact on treatment outcomes. Additionally, it summarizes data from the literature on paraneoplastic syndromes that precede PDAC diagnosis to highlight unusual symptoms that may initially seem unrelated to cancer located in the pancreas. While these factors may not be immediately linked to cancer diagnosis and prognosis, they could serve as useful indicators for earlier detection of this neoplasm.

A search of data was conducted for relevant articles using the PubMed and Google Scholar databases using keywords and combinations such as “diabetes”; “pancreatic cancer” and “diabetes”; “pancreatic ductal adenocarcinoma” and “diabetes”; “depression” and “pancreatic cancer” and “pain”; “paraneoplastic syndrome” and “pancreatic cancer”. The references of the articles used were also searched for additional appropriate studies. All the articles considered were selected without restriction at the time of publication or study type, including case–control and cohort studies, clinical trials, meta-analyses, traditional reviews, and systematic reviews. The search was restricted to only English-language literature.

## 2. Pain in Pancreatic Cancer

### 2.1. Underlying Biological Mechanism of Pain in Pancreatic Cancer

Research conducted between 2016 and 2018 by The Pancreatic Cancer Action Network showed that 93% of patients reported pain related to PC diagnosis, with 83% classifying it as moderate to severe [6]. Pain is the second most common symptom for patients with tumors in the pancreatic tail and body and the third most common for those with tumors in the pancreatic head. This means that while pain is a common manifestation of PDAC itself, there are also some uncommon causes that are not directly related to the tumor mass (such as infiltration or metastases) but rather arise from its humoral and cytokine activity. Moreover, pain can also be caused by a paraneoplastic syndrome [7]. Because of that, pain remains a significant factor to consider within the scope of this study, which aims to further investigate symptoms that may precede the diagnosis of PDAC. In this context, pain was identified as the most prevalent pre-diagnostic symptom reported by patients with PDAC, estimated at approximately 60% according to a recent survey study [8]. Patients with PC frequently experience severe abdominal pain, which can be caused by factors such as tissue destruction, inflammation, ductal blockage, and infiltration. The pathophysiology of pain in PC has been linked to inflammatory cells and various neurotransmitters, including glutamate, substance P, nerve growth factor (NGF), and calcitonin gene-related peptide. The severity of pain correlates with increased NGF secretion, driven by macrophage infiltration into pancreatic tumors. These tumors primarily cause occlusion of the main pancreatic duct, resulting in increased intraductal pressure and a deficiency of pancreatic exocrine enzymes, leading to malabsorption and postprandial pain [8]. Furthermore, it has been observed that as many as 80% of PC patients experience neuropathic pain [9]. Neuropathic pain, resulting from conditions like PDAC-associated neuropathy, is caused by damage to peripheral and central nerves, including spinal nerves and the thalamus. PNI influences the development of the tumor microenvironment (TME), where cells release factors that promote PNI and neuropathic pain. These factors include pro-inflammatory cytokines such as interleukin 6 (IL-6), interleukin 1β (IL-1β), and tumor necrosis factor-α (TNFα) [10]. Additionally, mention should be made of neurotransmitters such as substance P, neurotrophins, and axonal guidance molecule slit guidance ligand 2 (SLIT2). Neuropathic pain is also linked to hyperglycemia, the presence of chemokines, and matrix metalloproteases [10,11,12,13].

In a research study conducted by Hirth et al., an RNA interference screen was executed targeting chemokines in mouse PDAC cells and sensory neuron co-cultures. Two chemokines, CCL21 and CXCL10, which are produced by sensory neurons, interact with the PDAC cell-expressed receptors CCR7 and CXCR3, thereby encouraging cancer cells to migrate towards neurons. This phenomenon results in neural remodeling characterized by nerve fiber hypertrophy in tumor-bearing mice. Furthermore, elevated rates of cancer-associated pain were correlated with higher expression levels of CXCR3 and CCR7 in human PDAC samples [14].

**Figure 1:**
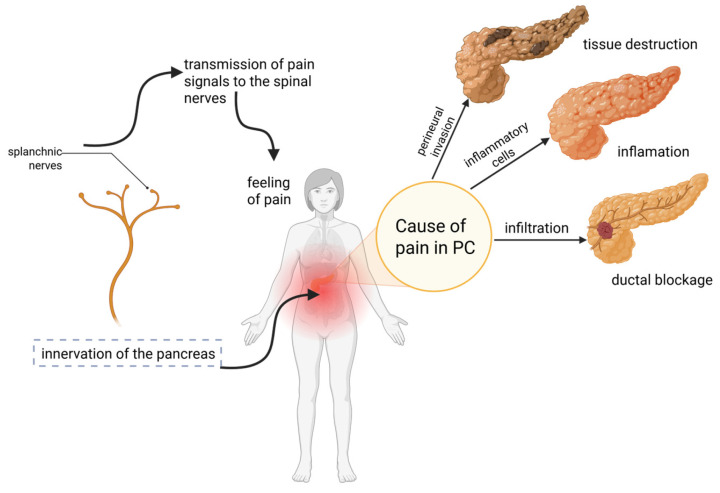
Biological mechanism of pain in PDAC. Perineural invasion leading to tissue destruction, the presence of inflammatory cells, and infiltration resulting in ductal blockage create pain signals. The splanchnic nerves, which innervate the pancreas, transfer pain signals to the spinal nerves. As a result, pain signals are transmitted, for example, to the thalamus, where the information is being processed and results in the perception of pain [8,10]. Created with BioRender.com. Włoszek, E. (2025). https://BioRender.com/606r6tc, Accessed on 24 September 2025.

The mechanisms of pain in PDAC with a cause-and-effect relationship are depicted in Figure 1.

### 2.2. Influence of Pain on Prognosis and Treatment

One-third of cancer patients experience insufficient pain management. Moreover, compelling evidence links mental distress, mood disorders, and anxiety to cancer pain. Pain and depression frequently occur together, disrupting functional capacity and making daily activities challenging. Their co-occurrence may exacerbate physical and psychological symptoms. Generally, the presence of pain in PC patients is associated with impaired survival [15,16]. It has been observed that the median survival time for patients without pain was 21.5 months, and for those with mild pain, it was 15.0 months; however, for patients experiencing moderate-to-severe pain, the median survival time was 10.0 months [7]. Severe pain was most often reported in cases of neural hypertrophy in chronic pancreatitis and pancreatic adenocarcinoma [17].

An article authored by Kelsen et al., published in 1997, demonstrated an unfavorable correlation between pain and survival outcomes. Furthermore, the presence of pain adversely affects the likelihood of surgical resection. Patients with operable pancreatic cancer who present with pain, even those whose examinations indicate a potential for resectability, exhibit a higher probability of recurrence and a diminished chance of survival compared to their pain-free counterparts [18].

A study by McNearney et al. reported that pain was most frequently experienced by patients whose PC had spread to the liver and peritoneum. Furthermore, patients with pain from PC prior to diagnosis experience a higher incidence of metastasis to the liver, peritoneum, and other gastrointestinal organs. Experiencing pain before the diagnosis was associated with a higher incidence of significant impairment in completing daily chores and often led to feelings of fatigue [19].

A study conducted by Grahm et al. indicated that patients with tumors located in the body or tail of the pancreas experienced a higher level of pain compared to those with tumors situated in the head of the pancreas. The stage or size of the cancer could not elucidate these relationships. Fifty-three percent of patients reported experiencing little to no pain at the time of diagnosis; however, this number decreased over time [20].

Tao et al. reported that high-intensity focused ultrasound (HIFU) combined with Gemcitabine and Oxaliplatin significantly alleviated pain among elderly patients diagnosed with middle to advanced PC. The Clinical Benefit Rate (CBR) was recorded at 68.4%, encompassing pain relief. The statistical significance associated with pain relief was indicated as *p* < 0.01. It is posited that HIFU’s capacity to induce coagulative necrosis in tumor tissues contributes to pain alleviation. This mechanism may interfere with pain pathways and reduce nerve irritation resulting from tumor progression [21].

A study to evaluate the safety and efficacy of combining focused ultrasound (FUS) with chemotherapy in patients with advanced pancreatic cancer was conducted by Bennett et al. It was found that this combination therapy led to significant pain relief. It is thought that FUS reduces tumor-induced nerve irritation by causing coagulative necrosis in tumor tissues. In general, the combination of chemotherapy and FUS was well tolerated, with few cases of serious side effects [22].

It has been shown that celiac plexus neurolysis (CPN) is effective in relieving pancreatic cancer-related pain. Puli et al. declared that overall pain relief from CPN was achieved in 72.5% of patients. Moreover, patients undergoing CPN experienced significant reductions in opioid consumption, leading to a reduction in opioid side effects. Pain relief began within 24–48 h after the procedure and lasted for several weeks to months. The most common adverse effects were transient diarrhea and hypotension [23].

## 3. Depression in Pancreatic Cancer

Depression is increasingly recognized in the general population, with a prevalence of about 6%, but it is significantly more common in oncology patients, in up to 8–24% of cases [24,25]. Major depressive disorder (MDD) occurs up to seven times more often in patients with cancer than in the general population [24]. A correlation between depression and cancer has been reported, although it was never clear whether this results from the difficulty of diagnosis and the uncertainty of prognosis or from the neoplastic process itself. The hypothesis that anxiety and depression themselves could have cancerogenic effects was disproved in a meta-analysis which pointed out that, instead, PDAC can be a reason for psychiatric symptoms [26]. Almost a century ago, one study of clinical reports reported psychological symptoms, namely depressed mood, anxiety, sleep disturbance, and a persistent sense of serious physical decline, to be the first symptoms of PC [27].

Among gastrointestinal cancers, pancreatic neoplasms have the highest depression rate, with prevalence estimates ranging from 38% to 45% [28]. Research from the Mayo Clinic showed that 76% of patients with PC had anxiety and depression symptoms, in comparison to 20% of patients with other neoplasms [29,30]. Based on a US-based healthcare database, an extensive study of 62,450 patients reported that 10,220 (16.4%) were diagnosed with depression before PC, and in 8130 (13%) cases, diagnosis of depression was made following that of PC, mainly in the first 6 months after diagnosis [31]. Some studies suggest that depression can significantly precede PDAC, with psychiatric symptoms appearing several years before somatic symptoms, in some cases more than three years prior [29,32]. Although anorexia, weight loss, and exhaustion are used to define depression in physically healthy people, in cancer patients, the psychological or cognitive symptoms of depression, like hopelessness, loss of self-esteem, anhedonia, guilt, and suicidal thoughts, are used to make a diagnosis [33]. The most common manifestations of depression in PDAC are psychomotor retardation, loss of energy, and feelings of worthlessness [24].

**Figure 2:**
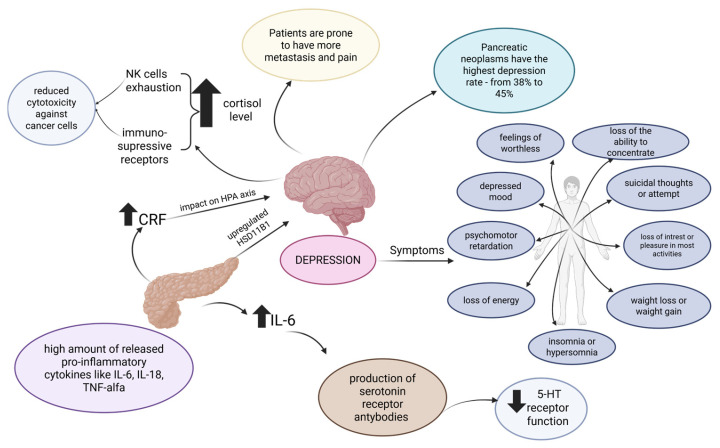
Depression in pancreatic cancer combines biological mechanisms and clinical features. Elevated pro-inflammatory cytokines, cortisol imbalance, and serotonin receptor dysfunction contribute to the onset of depressive symptoms. Patients present with loss of energy, depressed mood, feelings of worthlessness, psychomotor retardation, loss of the ability to concentrate, sleep disturbances, and weight changes [41]. The prevalence of depression in pancreatic cancer reaches 38–45% and is linked to higher pain burden and metastasis. The upward arrow indicates an increase in level, while the downward arrow represents a decrease in level. Created with BioRender.com. Włoszek, E. (2025). https://BioRender.com/1k9s2og, Accessed on 24 September 2025.

A dysregulated immune response is correlated with depression. Elevated pro-inflammatory cytokines like IL-6, IL-18, and TNF-alpha may stimulate the release of corticotropin-releasing factor (CRF) and impact the hypothalamic–pituitary–adrenal axis, resulting in, for example, higher cortisol levels [34]. Moreover, 11-hydroxysteroid dehydrogenase type-1 (HSD11B1), which elevates cortisol production, is upregulated in PDAC. Hypercortisolemia is correlated with the mRNA levels of eleven immunosuppressive receptors in PDAC and NK cell exhaustion, resulting in reduced cytotoxicity against cancer cells [35]. IL-6 supports tumor cells’ proliferation and creates an anti-apoptotic environment [36]. Moreover, elevated IL-6 plasma levels in cancer patients with depression may be correlated with the production of serotonin receptor antibodies by tumor cells [34,37,38]. These antibodies, as an alternative receptor for serotonin, can reduce total 5-hydroxytryptamine (5-HT) receptor functional activity, resulting in depressive illnesses [38]. An elevated activity of indolamine 2,3-dioxygenase is also considered a potent factor related to the increased incidence of depression in PC patients. This kynurenine pathway enzyme of tryptophan catabolism leads to a reduction in serotonin levels and the accumulation of cytotoxic metabolites in the brain [39,40]. The connection between the biological mechanisms of depression and PDAC is depicted in Figure 2.

There is a high mutual presence of mental disorders such as depression and anxiety alongside cancer, and the risk of developing mental disorders is higher in cancer patients before and after diagnosis than in the general population. Psychiatric symptoms tend to occur within the year preceding the diagnosis of cancer [42]. Furthermore, some studies suggest that depression may be a feature of paraneoplastic syndrome associated with PC [43]. However, evidence for an association between the pre-existence of these conditions and the risk of PDAC is not sufficient to support the use of psychiatric symptoms as indicators for PC screening [44]. In addition, researchers use different diagnostic tools to assess psychiatric symptoms, such as depression and anxiety, and the results of their studies differ significantly. Therefore, this makes it difficult to compare and summarize available data regarding the time relationship between depression and PC [24]. In patients with evidence of mental disorders and somatic symptoms such as abdominal pain, weight loss, and fatigue, it is crucial to exclude organic diseases, including PC, before making a psychiatric diagnosis [43,44]. Depression is a risk factor for decreased compliance in oncological patients. Thus, it affects many factors important for treatment, such as lower cognition and immune deficiency, fatigue, pain, and loss of appetite [24,45,46]. It was observed that patients with PDAC present with greater cognitive difficulties accompanying depression than patients diagnosed with depression in other cancers [46]. Depression significantly impairs quality of life (QoL) and treatment adherence, and it may reduce OS [24,47,48]. However, one meta-analysis showed that it predicted mortality, but not cancer progression. Mortality rates were up to 25% higher in patients experiencing depressive symptoms and up to 39% higher in patients diagnosed with major or minor depression [49]. Depression or anxiety diagnosed before cancer in patients with metastatic PDAC lowers OS and the chances of receiving chemotherapy [24,50]. Additionally, patients with depression are prone to have more metastases and pain than non-depressed patients. That is why pain is also considered a worse prognosis factor, and it has a role in causing depression [40,51,52].

New-onset depression is especially associated with lower survival; that is why patients with PDAC newly diagnosed with the disorder should be closely monitored and offered solutions to improve their treatment [53]. The choice of antidepressant therapy should rely on the main target symptoms in each patient; the patient’s overall clinical presentation, cognitive functions, and other psychiatric conditions; potential interaction with other co-administered drugs; and the need to avoid side effects caused by antidepressants [54]. Selective serotonin reuptake inhibitors (SSRIs)—fluoxetine, sertraline, paroxetine, fluvoxamine, citalopram, and escitalopram—remain the gold-standard treatment for depression due to better tolerability and fewer side effects compared to other antidepressant drugs [55,56]. However, for patients who do not respond to first-line medication, other agents may be prescribed, like serotonin-norepinephrine reuptake inhibitors (SNRIs), such as venlafaxine and duloxetine, or tricyclic antidepressants (TCAs) [56].

The impact of antidepressant therapy on cancer patients was assessed in several studies. It has an acceptable safety profile, and the most common adverse effects are nausea, vomiting, abdominal pain, and dry mouth [57]. Chen et al. showed that fluoxetine not only diminished extracellular signal-regulated kinase (ERK)/NF-κB-modulated anti-apoptotic and invasive potential but also promoted apoptosis through extrinsic or intrinsic pathways in hepatocellular carcinoma (HCC) cells in vitro [58]. In Di Rosso et al.’s study, fluoxetine and sertraline restored the antitumor immune response in lymphoid cells [59]. Jia et al. demonstrated that in pancreatic tumors, mirtazapine and fluoxetine do not affect neoplasm growth in human pancreatic carcinoma xenografts in nude mice. However, in the mirtazapine group, food intake and nutrition were significantly improved compared to the fluoxetine group. The effect of these drugs on the growth of PC in nude mice was not reported [60]. According to the results, mirtazapine could be an option to improve food intake in PC patients with depression and weight loss, but further investigation should be conducted. Antidepressants may reduce cortisol levels by regulating the function of the glucocorticoid receptor and the HPA axis [61]. Given that elevated cortisol can influence the pancreatic tumor microenvironment, these effects may have potential therapeutic implications. However, further studies are necessary to fully evaluate and understand their impact.

One case report was published describing a patient with depression who achieved long-term complete remission in advanced PC with liver metastases after receiving first-line chemotherapy with gemcitabine and nab-paclitaxel. Psychotherapy also positively influenced the patient’s mental health, which could be one of the factors contributing to remission [62]. Such psychological support by mental health professionals can improve QoL and may impact the psychological component of some symptoms, such as pain, anxiety, fatigue, nutrition, or gastrointestinal issues [63]. Another psychological factor that is responsible for increased anxiety in patients with PDAC and that needs to be taken into account in therapy is fear of recurrence, regardless of possible remission [45]. That is why patients with PDAC may better cope with the disease and therapy if they have greater internal resources and support from the beginning of the treatment. In Woo et al.’s randomized study (2019), early palliative care (EPC) focusing on pain and depression improved early pain control and QoL, although it did not significantly reduce depressive symptoms in patients with advanced PC and biliary tract cancer [64]. Another study, the multicenter, randomized phase III EPIC trial (NCT02853474), assessed whether early integration of EPC with standard oncological care (SOC) could improve OS in patients with metastatic upper gastrointestinal cancers, including PC. However, the results did not show differences between these two groups. Although the QoL was not improved, future studies should still focus on and explore the beneficial effect of EPC [65].

## 4. Diabetes in Pancreatic Cancer

### 4.1. Epidemiology and Characteristics

The Global Burden of Disease Study in 2021 estimated that there are approximately 529 million patients with diabetes mellitus (DM), representing a global diabetes prevalence of around 6%, with type 2 DM accounting for 96% [66]. As the American Diabetes Association states in the classification of DM, there are four main categories:

1. Type 1 DM (T1DM) (autoimmune β-cell destruction);
2. Type 2 DM (T2DM) (non-autoimmune progressive loss of adequate β-cell insulin secretion);
3. Specific types of DM due to other causes (this type includes diseases of the exocrine pancreas);
4. Gestational DM [67].

In a comprehensive cohort study, no association was identified between T1DM and PDAC, thereby corroborating the hypothesis that chronic hyperinsulinemia associated with long-standing T2DM plays a significant role in the development of PDAC [68]. There are two predominant types of diabetes mellitus diagnosed among patients with PDAC. Recognizing these distinct types may assist in establishing variances in subsequent diagnostic procedures. They include

(1) T2DM [69].
(2) PDAC-associated type 3c diabetes mellitus (T3cDM)—a state of hyperglycemia as a result of pancreatic dysfunction, which can be detected in the early stage of PDAC, even if the tumor is not yet visible in imaging studies [70].

T2DM constitutes a significant risk factor for the development of PDAC. This risk is notably amplified in individuals with a long-standing history of the disease, specifically those with a duration exceeding three years, resulting in an approximate twofold increase in the likelihood of PDAC. The highest risk is particularly prevalent among older patients, typically in their fifth decade of life, especially those who exhibit concurrent obesity or metabolic syndrome, as well as a family history of T2DM [69,71].

Numerous studies propose that the presence of nausea, vomiting, and diarrhea (NOD) associated with pancreatic ductal adenocarcinoma (PDAC), along with additional weight loss preceding cachexia, constitutes a paraneoplastic syndrome. This condition should be regarded as a “red flag,” particularly in older patients (aged 60 years and above), thereby underscoring the necessity for PDAC screening within this demographic [72].

T3cDM is believed to impact approximately 1% of patients diagnosed with NOD who are over the age of 50; however, it is highly likely to be underdiagnosed [73]. Data estimates that T3cDM is associated with PDAC in 30% of patients [74].

The evaluation of diabetes prevalence in PDAC revealed a significantly higher occurrence of diabetes in this group compared to the general population, unlike any other malignancy [74]. One half of patients diagnosed with this tumor met the clinical criteria for diabetes, and 85% had elevated fasting blood sugars, with a diagnostic window of diabetic symptoms before any other symptoms of cancer in about 25% of those patients [71,74].

Available data suggest that the presence of PDAC can be either a result or a cause of diabetes; especially new-onset disease may be indicative of concomitant cancer [75]. The study by Pannala et al. (2008) shows that 57% of patients with NOD and PDAC experienced an improvement in the DM course after pancreaticoduodenectomy, indicating that NOD results from the tumor [76]. On the other hand, patients diagnosed with NOD within three years have less than a 1% chance of developing PDAC, with most of this risk appearing within the first year [71,75,77,78].

DM associated with chronic non-malignant diseases of the exocrine pancreas is not a risk factor for developing PDAC. However, the conditions that cause insufficiency of the exocrine pancreas, such as chronic alcohol-induced pancreatitis, which increases the risk by approximately 4%, may play a role in the development of PDAC [69].

### 4.2. Underlying Biological Mechanism

**Figure 3:**
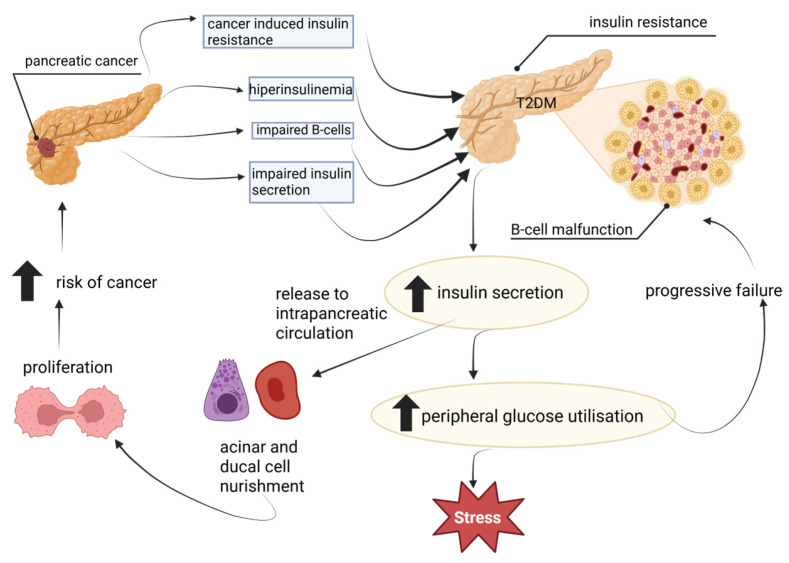
Bilateral relationship between diabetes and PDAC. Own elaboration. The upward arrow indicates an increase in level. Created with BioRender.com. Włoszek, E. (2025). https://BioRender.com/z2u7xg2, Accessed on 24 September 2025.

T2DM is known to increase the risk of PDAC, but its relationship with this type of cancer may be more complex, suggesting that T2DM could also be both an early symptom and a result of this neoplasm [79,80]. The potential mechanisms of the bilateral relationship between DM and PDAC are illustrated in Figure 3.

The tumor microenvironment (TME) is greatly influenced by metabolic abnormalities that promote inflammation and immunosuppression, which in turn leads to tumor progression. DM can affect these metabolic changes by altering glucose, amino acid, and lipid metabolism [81]. Dyslipidemia is a risk factor of PDAC [82], and interestingly, statins may potentially reduce PC risk and improve survival in patients with of both metabolic syndrome and PC [83].

Hyperglycemia associated with DM significantly stimulates proliferation, which in turn promotes carcinogenesis; due to the Warburg effect, glycolysis is sufficient for survival during nutritional deficiency [84]. An in vitro study by Zhang et al. (2022) stated that hyperinsulinemia modulated pathways associated with protein translation, MAPK-ERK signaling, and PI3K-AKT signaling, which were altered in epithelial cells and subsets of immune cells [85]. Insulin binding to its receptor has the capability to activate downstream MAPK and PI3K pathways, which results in cellular proliferation [86].

A study by Zhang et al. (2023) has shown that hyperinsulinemia initiates PC by increasing the production of digestive enzymes and inflammation via acinar insulin receptors [87]. Activation of intracellular receptors—IR, IRS1, and IRS2—caused by abnormally increased expression of docking peptides in PDAC cell lines subsequently activates the PI3K signaling cascade [86,88].

Moreover, hyperglycemia is connected to carcinogenesis by inducing lipotoxicity and pathways such as glycosylation, autoxidation, oxidative phosphorylation, the glucosamine pathway, and the Hip-po-Yes-associated protein (YAP) pathways [89,90], with the dysfunction of these pathways increasing reactive oxygen species (ROS) and as a result affecting DNA in β-cells, causing instability [91]. Furthermore, epidermal growth factors (EGFs) and their receptors (EGFRs) are promoted by hyperglycemia, which causes increased angiogenesis [92].

Another factor influencing cancer cell biology is hyperinsulinemia, a compensatory response to hyperglycemia. Both of these conditions significantly contribute to an increased mortality rate in PDAC [93]. The mechanism underlying this phenomenon is that, in the course of DM, patients develop insulin resistance, which signifies an increase in serum insulin levels accompanied by a simultaneous decrease in tissue sensitivity to insulin [93]. Islet amyloid polypeptide (IAPP) constitutes a significantly elevated factor that may contribute to the development of insulin resistance in skeletal muscle cells, particularly in patients diagnosed with PDAC [94]. PDAC cells have the ability to facilitate the release of IAPP from islet cells. However, hopes for IAPP to act as a biomarker for the diagnosis and identification of PDAC have been dismissed due to the absence of its previously reported tumor suppressor function, indicating that IAPP does not increase the risk of PDAC in diabetic patients [95].

Furthermore, the S-100A8 peptide derived from PDAC induces insulin resistance and is suggested by some studies to be a potential biomarker for the early detection of PDAC [96].

### 4.3. Available Solutions for Screening

In light of the prevalent issue of delayed diagnosis and the significant mortality associated with PDAC, it is imperative to identify a non-invasive approach for the early detection of this malignancy [97]. According to the guidelines set forth by the U.S. Preventive Services Task Force, it is advised that screening of the general population, including asymptomatic NOD patients, for PDAC should be avoided [98,99]. Patients with mutations associated with PC, specifically BRCA1 and BRCA2 mutations, represent the sole high-risk demographic recommended to undergo regular screening for PDAC, as stated by the American Society of Gastrointestinal Endoscopy [100]. Despite the increased risk of PDAC in individuals with NOD, there are presently no specific recommendations for PDAC screening within this population. A case–control study indicates that hyperglycemia typically presents approximately 36 to 30 months prior to the diagnosis of PDAC and experiences a rapid decline during the 12 to 6 months preceding the diagnosis [101]. Therefore, it is essential to quickly identify NOD patients who are at a high risk of PC-associated diabetes mellitus (PaCDM) [69]. The risk factors associated with PaCDM encompass weight loss, advanced age (over 50 years), a familial history of PC, the presence of gallstones, episodes of pancreatitis, and a decline in hyperglycemia and HbA1c levels [43,69,77,102]. The presence of recognized signs and symptoms should prompt clinicians to consider surveillance for PDAC [102,103,104].

A study conducted by Sharma et al. sought to develop a predictive model, known as the END-PAC score, for the purpose of evaluating the risk of PC among patients with NOD [105]. This model, which incorporates three variables—weight change, blood glucose change, and age at diabetes onset—was further validated in subsequent studies [106,107]. Currently, the research focuses on identifying effective screening methods to distinguish patients with NOD and PaCDM from other forms of DM [75]. The ideal screening test should possess characteristics that ensure its validity, consistency, tolerability, ease of application, and cost-effectiveness. A multitude of methods have been proposed as potentially effective, including blood tests such as the concentration of pancreatic polypeptide, glucose-dependent insulinotropic peptide, antibodies against apolipoprotein-AII isoforms, serum lipid profiles, circulating levels of galectin-1, osteonectin concentration, and a panel of biomarkers that includes tissue factor pathway inhibitor, tenascin, and carbohydrate antigen 19-9 (CA19-9), as well as the expression of microRNAs and tRNAs and circulating free DNA in plasma, alongside various machine learning models, risk prediction models, and urine biomarkers that all have been summarized and compared in Table 1 [108,109,110,111,112,113,114,115,116,117,118,119,120,121,122].

However, it is imperative to identify those suitable for integration into standard clinical practice. To date, none of these concepts have been validated and incorporated into any clinical protocols for patients with NOD. Currently, CA19-9 is recognized as the only serum biomarker for PDAC that has received approval from the Food and Drug Administration in the United States, and it is utilized in the diagnosis and monitoring of the disease. Nonetheless, its effectiveness in PDAC screening is constrained by low specificity (90%) and sensitivity (70%) [127]. The gold standard for diagnosing PDAC is contrast-enhanced computed tomography (CT). However, it is relatively expensive and carries ionizing radiation risks and complications, which render it an unsuitable screening method [128]. A recently published study indicated that non-contrast CT enhanced by an artificial intelligence (AI) algorithm may be a better option for screening patients, as it has fewer side effects compared to contrast-enhanced CT and boasts a very high specificity of 99% in identifying the seven most common pancreatic lesions [129]. A study by Zeng et al. (2022) identified 44 genes shared between PC and T2DM [123]. Notably, the discovery of the hub gene S100A6 in both PC and T2DM may serve as a predictive biomarker for detecting PDAC in T2DM patients [123].

**Figure 4:**
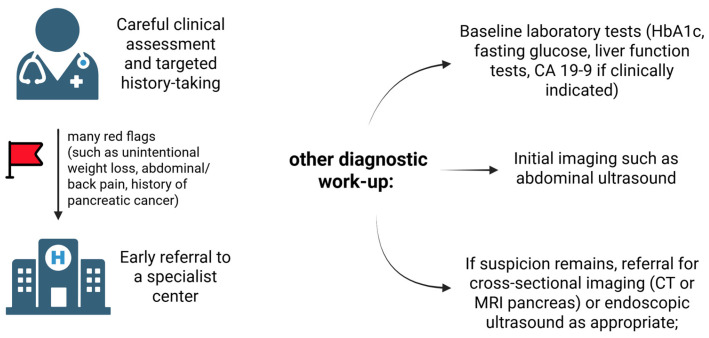
Diagnostic approach for suspected pancreatic issues. Own elaboration. Created with BioRender.com. Fudalej, M. (2025). https://BioRender.com/mdt4esn, Accessed on 24 September 2025.

Considering that newly diagnosed DM heightens the risk of PDAC and may act as an early indicator of PDAC in conjunction with other symptoms, the implementation of a clinical screening protocol for these patients is imperative. Consequently, future studies should concentrate on the development and validation of such guidelines. Nevertheless, in patients over 50 with NODM, the primary care provider (GP)’s history-taking should intentionally go beyond just glycemic control to include symptoms like unintentional weight loss, abdominal or back pain, and a family history of pancreatic or other cancers. By incorporating these factors into the clinical interview, GPs can identify patients who need further diagnostic testing, potentially leading to earlier detection of pancreatic cancer, which can improve the prognosis. The suggested diagnostic process in cases of suspected PDAC is depicted in Figure 4. 

### 4.4. Influence on Prognosis and Treatment

Patients diagnosed with PDAC who also had an asymptomatic stage of DM were found to have a better prognosis than those who had symptoms of DM at the time of cancer diagnosis. This finding suggests that early detection of DM can be crucial for enhancing PDAC screening [130]. In our retrospective study of 175 patients with PDAC, those with DM who received palliative chemotherapy had a significantly higher median OS compared to those without DM (18 months vs. 13 months, respectively, *p* < 0.034). No difference in survival was detected between the non-DM and DM groups in the adjuvant cohort [131]. Nevertheless, in the randomized controlled trial, the multivariable-adjusted HR for mortality comparing participants with diabetes to those without was 1.52 (95% CI = 1.14–2.04, *p*-value < 0.01) [132].

Among the various factors that contributed to the increased mortality rate was the duration of the disease (pertaining to T2DM—over 5 years, compared to new onset) [133]. There is no consensus in the literature regarding the influence of DM on post-operative OS and mortality rate (MR) for patients with PDAC, except for an increased MR in those with long-standing T2DM (over 5 years). A study by Li et al. (2015) indicated that OS was reduced by 1.2 months for patients with DM, and for those who underwent surgery, it decreased by as much as 11 months [134]. Additionally, a study by Deo et al. showed that DM had no impact on MR, morbidity, or duration of hospitalization in patients undergoing pancreatoduodenectomy. However, it significantly affected the odds ratio (OR) and 3- and 5-year survival, with no impact on 1-year survival [135]. Studies have shown that patients with DM also had elevated CA19-9 levels, significantly larger tumor sizes (>2 cm), and higher rates of lymph node involvement, as well as perineural and/or lymphovascular invasion [134,136].

Treatment with metformin could lower the risk of developing PDAC by 18%, making it one of the best options for DM therapy. However, the data in the literature is not clear [69]. The mechanism of the antitumoral properties of metformin consists of modulating histone acetyltransferases (PCAF, p300, CBP) and SIRT1 expression, leading to cancer cell apoptosis [137]. On the other hand, a cohort study conducted in Korea indicates that women with diabetes using metformin are at a higher risk of PC than women with diabetes not using metformin (this risk is not elevated in men) [138]. A recent observational study conducted by the PREOPANC randomized controlled trial group found that patients using metformin exhibit specific antitumoral immune responses in upfront resected PDAC, emphasizing that this phenomenon does not occur in tumors treated with neoadjuvant chemoradiotherapy [139]. Immunological changes involve a decrease in protumoral M2 macrophages and an increase in tumor-resolving dendritic cells, which could result in prolonged survival [139]. A meta-analysis concluded that there was a significant improvement in survival (HR = 0.86, 95% CI 0.76–0.97; *p* < 0.05) in the group of patients with PDAC receiving metformin compared to controls [140]. Metformin improved survival in patients after resection and in those with locally advanced tumors, but it did not have the same effect in patients with metastases, indicating that the effectiveness of metformin correlates with tumor stage [140,141]. On the other hand, an umbrella review by Nowicka et al. (2023) showed that the quality of evidence in meta-analyses connecting metformin use to decreased pancreatic cancer mortality is low due to various types of bias in retrospective studies [142]. Another study stated that the association between metformin and pancreatic cancer survival has been overemphasized in cohort studies due to immortal time bias [143]. Nevertheless, there is currently no clear data supporting the routine use of metformin as an adjuvant therapy for PC, although it may improve OS in cases of resectable tumors [144].

Other anti-diabetic drugs, including sulfonylureas, thiazolidinediones, dipeptidyl peptidase 4 (DPP-4) inhibitors, glucagon-like peptide-1 receptor agonists (GLP-1 RAs), and sodium-glucose co-transporter 2 (SGLT-2) inhibitors, have been reported to influence PDAC treatment outcomes [145]. A study conducted by Szymczak-Pajor et al. revealed that glimepiride and glibenclamide, but not gliclazide, induced pancreatic cancer cell death through mechanisms involving increased Ca2+ intracellular levels, along with CASP-3 and p53 [146]. Pioglitazone acts as a peroxisome proliferator-activated receptor γ agonist, and its antitumoral effect involves promoting an excessive production of ROS, which inhibits cell proliferation and induces cell death [147]. Moreover, thiazolidinedione modulates the E-cadherin/beta-catenin system in a human pancreatic cancer cell line, which makes it potentially clinically useful as a cytostatic anti-cancer agent [148].

Despite numerous concerns, there is no definitive evidence that DPP-4 inhibitors raise the risk of PC [149]. A study by Li et al. finds that saxagliptin, a DPP-4 inhibitor, significantly induces β-cell proliferation and upregulates the expression of proliferation-related factors by increasing stromal cell-derived factor-1α (SDF-1α) and states that SDF-1α could be a therapeutic target for β-cell regeneration [150].

Some studies link GLP-1 RAs and their risk of pancreatitis to an increased risk of PC, but the latest studies show no clear evidence of risk for pancreatitis associated with this group of anti-diabetic drugs [151]. Moreover, studies have shown that GLP-1RAs are associated with a reduced incidence of PC in patients with T2DM [152,153]. Liraglutide inhibited the growth of PC and promoted apoptosis by suppressing the PI3K/Akt and ERK1/2 signaling pathways [154]. A recent study published by Cencioni et al. (2025) resulted in the proposal of novel therapeutic options for PDAC patients with metabolic syndrome, stating that semaglutide prevented the higher dysmetabolism-dependent PDAC stromal deposition by reshaping pancreatic cancer-associated fibroblasts, reducing collagen proline hydroxylation, and additionally promoting T lymphocyte infiltration, which reduced tumor development [155].

Canagliflozin is a SGLT-2 inhibitor that exerts an antitumor effect by inducing early apoptosis and reducing the protein levels of PI3K, p-AKT, p-mTOR, and HIF-1α. Consequently, this impairs glycolysis, leading studies to suggest the potential role of the PI3K/AKT/mTOR signaling pathway in the treatment of PC [156]. Preclinical study results have shown that canagliflozin and dapagliflozin used in PC enhance the effects of gemcitabine chemotherapy, reduce cell proliferation, suppress glycolysis, and induce necrosis in tumors [157]. In an observational study involving patients with advanced, inoperable PDAC, the combination of dapagliflozin and chemotherapy showed good tolerability and indicated positive changes in body composition and plasma biomarkers [158].

A recent retrospective cohort study indicated that the cohort of patients diagnosed with PDAC who underwent surgical intervention comprised 75.9% non-diabetic patients and only 30% diabetic patients. The authors attributed this disproportion to comorbidities, malnutrition (with moderate or severe malnutrition associated with diabetes mellitus in 74.7% of cases), and a general decline in health among diabetic patients. It is noteworthy that this study also found that episodes of vomiting occurred more frequently among patients with DM [159].

## 5. Paraneoplastic Syndromes Preceding the Diagnosis of Pancreatic Cancer

Different categories of unusual symptoms that may occur before the diagnosis of PDAC, which should raise clinical suspicion and prompt oncological screening, include paraneoplastic syndromes that are compared in Table 2.

Trousseau’s syndrome is described as unexplained, spontaneous thrombophlebitis, linked to microangiopathy, that precedes the diagnosis of an occult visceral malignancy or appears at the time of the diagnosis [168]. Multiple overlapping mechanisms are involved in this phenomenon, such as tissue factor, thrombin, cancer procoagulant (cysteine proteinase), tissue hypoxia, carcinomas mucins, and oncogene activation [168,169,170]. Data on cancer incidence in a group of patients with unexplained venous thrombosis is not clear. A large meta-analysis concluded that screening for occluded visceral malignancy after idiopathic venous thromboembolism does not affect mortality rates. However, more cancer diagnoses were made shortly after the thrombotic event. Therefore, a risk population suitable for screening needs to be identified in further research [171]. The complexity of Trousseau’s syndrome’s pathophysiology might explain why it responds well to heparin, which has multiple moderating effects and has been shown in meta-analyses to produce the best treatment outcomes in cancer patients [172]. Trousseau’s syndrome can affect a patient’s prognosis by primarily causing thromboembolic events, such as multiple cerebral infarctions [173]. The connection between PDAC and Trousseau’s syndrome may be due to the excessive production of mucin by cancer cells, which is specific to adenocarcinomas and aggressive neoplasms and may contribute to thrombosis, which is often associated with this type of cancer. Mucin compounds play a key role in thrombotic events by interacting with the selectin family of adhesion molecules, leading to platelet activation and aggregation without involving thrombin [169].

Another paraneoplastic syndrome in PDAC involves panniculitis, which is defined as subcutaneous fat necrosis presenting as non-specific erythema tender nodules. It occurs concomitantly in 0.3–3.0% of pancreatic disorders such as pancreatitis, PC, and pancreatic pseudocyst [174]. Panniculitis appears as erythematous, ill-defined, red or brown nodules localized on the lower limbs and buttocks, rarely on the trunk and upper extremities [175]. The pathophysiological mechanism remains unclear; some data suggest the involvement of pancreatic enzymes. Conversely, some case studies report panniculitis without elevated pancreatic enzymes, indicating the possibility of alternative mechanisms [176]. It may present as a triad of pancreatic disease, panniculitis, and polyarthritis (PPP) syndrome, which can be a rare, early manifestation of yet undiagnosed pancreatic neoplasia, accounting for 11.9% of PPP syndrome cases [162,163]. Arthritis was present in 35% of cases with PC and PPP syndrome, associated with a worse prognosis due to a poor response to nonsteroidal anti-inflammatory drugs and corticosteroids and the rapid development of radiographic joint damage [164]. The diagnostic process for skin lesions should include imaging studies, skin biopsy, serum pancreatic enzyme levels, and detection of tumor markers [177]. Treatment should be directed at pancreatic neoplasms because the response of arthritis and skin lesions to steroids and nonsteroidal anti-inflammatory drugs is poor. However, the authors of a case report stated that the skin lesions responded well to octreotide, an inhibitor of pancreatic enzyme production [162].

To elaborate on rheumatologic paraneoplastic syndromes, another exceptionally rare phenomenon will be described: palmar fasciitis and polyarthritis syndrome (PFPAS). This condition manifests with inflammation of the palmar fascia and tendon sheaths, nodular thickening of the palms, and painful swelling of the hands. It also involves generalized arthritis primarily affecting the small joints of the fingers and wrists, leading to fibrosis and the rapid development of flexion contractures in the hands and wrists [178,179]. Those symptoms can resolve after corticosteroid treatment [180]. Nevertheless, the best treatment results for resolving PFPAS symptoms are obtained by treating the underlying malignancy with chemotherapy and/or surgery [181].

Paraneoplastic myopathies, including dermatomyositis and polymyositis, are quite rare in PC. As a result, many meta-analyses have only reported isolated cases [167,182]. It is suggested that the underlying mechanism of this paraneoplastic phenomenon involves an anti-cancer immune response triggered by similar antigens found in pancreatic tumors and regenerating muscle, but it has not yet been fully understood [167,183]. Previously mentioned case studies have ruled out drug-induced myopathy (especially well-known gemcitabine involvement (which is often included in PDAC chemotherapy) [184] and statin-induced myopathy [185], focusing on a possible paraneoplastic etiology that preceded the cancer diagnosis. Symptoms of idiopathic inflammatory myopathies according to the 2017 EULAR/ACR classification criteria include progressive, symmetric muscle weakness, especially of the proximal upper extremities, skin manifestations such as heliotrope rash, Gottron’s papules, Gottron’s sign, dysphagia, or esophageal dysmotility [186]. The diagnostic process in patients presenting with similar symptoms suggests performing a muscle biopsy, assessing serum auto-antibody levels, and electromyography to assess the clinical probability of inflammatory myopathy [167]. Treatment for a paraneoplastic etiology is similar to that for other inflammatory myopathies and mainly relies on glucocorticoids, which induce remission. Additional drugs that may be used include methotrexate, azathioprine, tacrolimus, ciclosporin, and mycophenolate mofetil [187]. It was described that myopathy-related rhabdomyolysis can be a first manifestation of pancreatic cancer; it is very uncommon and may lead to many complications and the need for renal dialysis [182].

Another very rare (affecting about 1 to 8 individuals per million annually) paraneoplastic manifestation of PDAC is transverse myelitis. It is a type of non-compressive myelopathy caused by autoimmune inflammation of the spinal cord, triggered by autoantibodies such as anti-glutamic acid decarboxylase 65 antibodies. These antibodies are associated with antigens of neoplasms that mimic those in neurons. Consequently, these antibodies target voltage-gated calcium and potassium channels, ganglionic acetylcholine receptors, and other specific components of the nervous system. Symptoms include neurological deficits (motor, sensory, and/or autonomic) depending on the level of a lesion in the spinal cord [188].

The occurrence of syndromes such as Trousseau’s syndrome or idiopathic pancreatitis can affect the clinical management by prompting appropriate oncological screening, which can result in early treatment and reduce the risk of dangerous thromboembolic events. 

## 6. Conclusions

Solutions for the early detection of PC represent a significant advancement in oncology that has been urgently required for decades to combat this highly malignant neoplasm. The identification of high-risk groups and early symptoms in PDAC, which may facilitate earlier diagnosis and treatment, is critical to this process, despite the limited patient population, which creates study limitations and may contribute to low evidence level and a possible publication bias. A comprehensive understanding of the biological mechanisms could aid in the pursuit of new biomarkers or potential screening tests, which are essential for enhancing patient outcomes. There is a pressing need for additional meta-analyses and multicenter, retrospective studies to investigate the prevalence and clinical characteristics of diabetes mellitus, depression, and pain that precede the diagnosis of PDAC.

## Figures and Tables

**Table 1:** Biomarkers in PDAC screening [114,116,117,118,123,124,125,126].

Biomarker	Clinical Use	Performance
CEA-19	approved by FDA	low specificity and sensitivity, used to monitor treatment response
glucose-dependent insulinotropic peptide	investigational	differential diagnosis with T2DM: lower in pancreatic cancer irrespective of the degree of glucose intolerance as compared to T2DM patients and healthy controls
pancreatic polypeptides	investigational	differential diagnosis with T2DM: lower in pancreatic cancer irrespective of the degree of glucose intolerance as compared to T2DM patients and healthy controls
tenascin C	investigational	locoregional recurrence-related poor prognosis marker, potential therapeutic target for PDAC
antibodies against apolipoprotein-AII isoforms	investigational	not useful for evaluation of clinical effect of CRT for PDAC; useful for assessment of pancreatic exocrine disorder; possible use in screening for the early stage of pancreatic cancer and, what is more, even identifying patients at risk for pancreatic malignancy
serum lipid profile	investigational	sensitivity and specificity over 90%, higher than those of CA 19-9, especially at an early stage, and comparable to established diagnostic imaging methods. What is more, selected lipid species indicate potential as a prognostic biomarker
circulating levels of galectin-1	investigational	sensitivity and specificity values similar to those of CA19-9; combination of galectin-1 and CA19-9 significantly improved their individual discriminatory powers; promising biomarker not only for detection but also for prognostics
osteonectin	investigational	possible PDAC screening marker that must be validated in prospective studies
LYVE-1, REG1A, and TFF1	clinical trials	triple protein test in urine, detecting early-stage PDAC: sensitivity of 96%, 100%, and 73.33%, respectively, and specificity of 100%, 82%, and 100%, respectively
circulating cell-free nucleatic acids: DNA, mRNA, non-coding RNA (miRNA and lncRNA)	clinical trials	high specificity for specific mutations, low sensitivity in early-stage cancers
hub gene S100A6	investigational	immune-related and potential therapeutic target for patients with PC and T2DM

**Table 2:** Paraneoplastic syndromes in PDAC.

Syndrome	Prevalence	Mechanism	Diagnosis and Management	Prognostic Significance
Trousseau’s syndrome	venous thromboembolism is 4- to 7-fold higher in patients with cancer than in those without cancer [160]	tissue factor, thrombin, cancer procoagulant, tissue hypoxia, and mucin overproduction by PDAC cells lead to platelet activation and aggregation without involving thrombin	effective prophylaxis and treatment (first line—low-molecular-weight heparin) reduces morbidity and mortality	high risk of thromboembolic events, worse prognosis
panniculitis	0.3–3.0% of pancreatic disorders	pancreatic enzymes and their activity in adipose tissue	imaging studies, skin biopsy, serum pancreatic enzyme levels, and detection of tumor markers, treatment of underlying malignancy, octreotide	could delay treatment of malignancy because of possible misdiagnosis as infection or rheumatologic disease [161]
PPP syndrome	PDAC is present in 11.9% of PPP syndrome cases [162,163]; arthritis is present in 35% of cases with PDAC and PPP syndrome [164]	pancreatic enzymes hyperactivity	imaging studies, skin biopsy, serum pancreatic enzyme levels, and detection of tumor markers, treatment of underlying malignancy	late diagnosis of underlying pancreatitis often results in a worse prognosis and inappropriate treatment [165]
PFPAS	extremely rare; 48 cases since first report described in the literature [166]	connective tissue growth factors and autoimmune mechanisms induced by the neoplasm	corticosteroids, treatment of underlying malignancy	unknown
dermatomyositis and polymyositis	14 per 100,000 [167]	anti-cancer immune response triggered by similar antigens found in pancreatic tumors and regenerating muscle	muscle biopsy, serum auto-antibody levels, electromyography, treatment with glucocorticoids	dermatomyositis is linked to a higher risk and worse survival rates compared to polymyositis; they both worsen the prognosis
transverse myelitis	1 to 8 individuals per million	autoimmune inflammation	treatment of the underlying malignancy	unknown

## Abbreviation

PC	Pancreatic cancer
PDAC	Pancreatic ductal adenocarcinoma
OS	Overall survival
NOD	New-onset diabetes mellitus
PNI	Perineural invasion
NGF	Nerve growth factor
HIFU	High-intensity focused ultrasound
CBR	Clinical benefit rate
CPN	Celiac plexus neurolysis
SLIT2	Slit guidance ligand 2
FUS	Focused ultrasound
MDD	Major depressive disorder
CRF	Corticotrophin-releasing factor
HSD11B1	11-hydroxysteroid dehydrogenase type-1
5-HT	5-hydroxytryptamine
QoL	Quality of life
TCAs	Tricyclic antidepressants
HCC	Hepatocellular carcinoma
GR	Glucocorticoid receptor
SSRIs	Selective serotonin reuptake inhibitors
SNRI	Serotonin–norepinephrine reuptake inhibitor
DM	Diabetes mellitus
T1DM	Type 1 DM
T2DM	Type 2 DM
T3cDM	Type 3c diabetes mellitus
TME	Tumor microenvironment
YAP	Hip-po-Yes-associated protein
ROS	Reactive oxygen species
EGFs	Epidermal growth factors
TGF-β	Transforming growth factor β
EMT	Epithelial-to-mesenchymal transition
PFK	Phosphofructokinase
RNR	Ribonucleotide reductase
IAPP	Islet amyloid polypeptide
OR	Odds ratio
DDP-4	Dipeptidyl Peptidase 4
SDF-1α	Stromal cell-derived factor-1α
GLP-1 RAs	Glucagon-like peptide-1 receptor agonists
SGLT-2	Sodium-glucose co-transporter 2
PaCDM	PC-associated diabetes mellitus
CA19-9	Carbohydrate antigen 19-9
CT	Computed tomography
MR	Mortality rate
AI	Artificial intelligence
PFPAS	Palmar fasciitis and polyarthritis syndrome
